# Assessing fiscal multipliers in times of crisis: evidence from selected CEE countries

**DOI:** 10.1007/s00181-023-02407-9

**Published:** 2023-03-27

**Authors:** Bogdan Muraraşu, Cristina Anghelescu, Robert Adrian Grecu

**Affiliations:** 1 Department of Money and Banking, Bucharest University of Economic Studies, 5-7 Moxa Street, 010961 Bucharest, Romania; 2 Bucharest University of Economic Studies, Bucharest, Romania

**Keywords:** Fiscal multipliers, Government measures, Emerging economies, Supply shocks, C11, C32, E62, E65, H50, H60

## Abstract

The COVID-19 pandemic proved to be an unprecedented socio-economic crisis in the last decades. More than three years after its outbreak, there is still uncertainty regarding its future evolution. National and international authorities adopted a prompt and synchronized response to limit the adverse effects of the health crisis, in terms of socio-economic damage. Against this background, this paper assesses the efficiency of the measures implemented by fiscal authorities in selected Central and Eastern European countries to ameliorate the economic repercussions of the crisis. The analysis reveals that the impact of expenditure-side measures is stronger than that of revenue-side ones. Additionally, the results of a time-varying parameter model indicate that the fiscal multipliers are higher in times of crisis. In view of the ongoing war in Ukraine, the related geopolitical turmoil and energy crisis, the findings of this paper are especially pertinent, given the need for additional fiscal support.

## Introduction

The ongoing public health crisis has simultaneously affected all sectors of the economy. Since its outbreak and its fast worldwide spread at the beginning of 2020, the COVID-19 pandemic has significantly changed the economic environment. Apart from its devastating effects on health and the staggering escalating death toll, the recent pandemic episode is also an unprecedented socio-economic crisis. From the social distancing measures imposed by authorities to self-induced ones, demand for certain services has dropped. Uncertainties are stemming from several sources induced by the pandemic and have the potential to lead to permanent changes in economic behaviour. Despite the introduction of vaccines against COVID-19, the pandemic threat still lingered in 2022 and is expected to weigh further on economic activity, as new and more contagious COVID-19 variants have emerged. Thus, as it might take time until the effects of vaccination campaigns become manifest, it is uncertain whether activity will return to normalcy as known before the pandemic. Even though at the beginning of 2022 the effects of the pandemic were gradually fading-off, a new threat to economic activity emerged. At the current juncture, the supply shocks induced by the Russian invasion in Ukraine have jeopardized the economic recovery of an already vulnerable global environment. The hikes in the prices of energy goods, previously triggered by the reopening of the economies, have recently been exacerbated by the international sanctions imposed to Russia. At the same time, the persistent disruptions in global production and supply chains, in tandem with novel episodes of increased risk aversion have led to high inflationary pressures as well. Given the fact that emerging economies in the geographical proximity of the war might be more exposed to the negative effects of the ongoing conflict and the necessary support measures of authorities might be relatively more consistent, the research is conducted for several Central and Eastern European (CEE) countries such as Bulgaria, Czechia, Hungary, Poland and Romania.

National authorities have tried to partially alleviate the effects of the pandemic crisis by introducing fiscal support measures. In some countries, the package of such initiatives and the variety of instruments were impressive. To these adds additional expenditure related to the public health crisis. At the same time, the European Commission has been coordinating a common European response. All these measures supported the national health systems and alleviated the socio-economic impact of the pandemic. Given the ongoing geopolitical crisis, such measures remain particularly relevant.

Economists all over the world have analysed the implications of the COVID-19 pandemic from a macroeconomic perspective. From the coronavirus impact on economic growth, labour market or certain industries, empirical evidence is gradually accumulating and bringing light on the effects of the unprecedented crisis. Even though there is no doubt the authorities’ response to the public health crisis was necessary, there is nevertheless scarce empirical evidence showing how efficient the implemented fiscal support measures proved to be.

Against this background, this paper focuses on the impact of the fiscal policies in times of crisis. As previously mentioned, in the recent period, the fiscal stance has suffered important reconfiguration. This was also true during the global financial crisis. As studies regarding the efficiency of the supportive measures related to the pandemic are scarce, this analysis concentrate particularly on the effects of fiscal measures adopted to alleviate the adverse effects of the health crisis.

The fiscal multipliers are a useful tool to quantify the effects of implemented support measures. The paper investigates the magnitude of fiscal multipliers and whether the pandemic affected the efficiency of the fiscal policies. Additionally, it provides empirical evidence for revenue and expenditure multipliers. Moreover, we consider the main sub-components of budgetary items. Furthermore, we illustrate the impact of support measures on economic activity. To this end, this analysis employs a variety of econometric tools, ranging from Bayesian Structural Vector Autoregressive (B-SVAR) models to Time-Varying Parameters Vector Autoregressive (TVP-VAR) models.

The rest of the paper is structured as follows. Section 2 provides an extensive summary of recent analysis focused on the quantification of fiscal multipliers in CEE countries and Sect. 3 presents some stylized facts regarding recent fiscal measures aimed at containing the effects of the adverse effects of the public health crisis. Section 4 describes data and methodology, while Sect. 5 summarises the main results. In a counterfactual exercise, the size of economic contractions that would have occurred in the absence of the adopted fiscal measures is also assessed. Section 6 concludes.

## Literature review

The novelty of the recent sanitary crisis made it difficult to assess its socio-economic effects. Under these circumstances, the COVID-19 pandemic has become a popular subject in economic literature. Since its beginning in early 2020, economists have made comparisons between the COVID pandemic and other sanitary crises or even the financial crisis. Jordá et al. (2020) compare European pandemics in order to assess their impact on the natural interest rate. As regards the comparison of the COVID pandemic with the 2008 global financial crisis, both Garrett and Gangopadhyaya (2020) and Li et al. (2021) find a higher adverse impact on the unemployment rate of the sanitary crisis. According to Gunay (2020), the effects of the financial crisis on the stock exchange are heftier than those prevailing during the pandemic. The less relevant literature is devoted to comparing the effectiveness of government support measures between the two recent crises.

At the current juncture, one research direction refers to the effectiveness of the measures adopted by the authorities in order to alleviate the adverse economic impact of the pandemic. Some of these initiatives have been extended given the negative economic effects stemming from the ongoing war in Ukraine, whose future evolution has become the main source of uncertainties for the outlook of the global economy. In the case of the fiscal policy, measures were adopted both on revenues and expenditure sides and implemented in a synchronized manner among European countries. Chudik et al. (2021) estimate a Global VAR model to assess the macroeconomic effects of discretionary fiscal policies in response to the COVID-19 pandemic. Their results show that fiscal policy is playing an important role in mitigating the effects of the pandemic and emerging markets are also benefiting from the synchronized fiscal actions globally. Asongu et al. (2021) conclude that there is a high heterogeneity as regards the response of economic activity to the measures adopted in the context of the sanitary crisis by analysing the economic policies mix among different economies. Croitorov et al. (2021) assess the macroeconomic impact of the COVID-19 pandemic in the euro area and highlight the heterogeneity of economic activity contraction across sectors, with a significant stronger negative impact on activities requiring human physical interaction. Weyerstrass (2021) and Prammer (2021) argue that the large-scale fiscal policy package implemented in Austria to cushion the economic impact of the containment measures contributed decisively to the stabilization of Austria’s public finances. Auerbach et al. (2021) document that the effects of U.S. government spending were stronger during the peak of the pandemic recession, but mainly in cities that were not subject to constraining “lockdown” measures. Medas et al. (2022) show that fiscal rules have been flexible during crises but have not prevented a large and persistent build-up of debt over time, with an implicitly negative impact on economic activity and highlight the need to further improve rule-based fiscal frameworks.

The impact of support measures on GDP can be inferred through the fiscal impulse (measured as the change in the primary structural balance). However, this paper does not focus only on the impact of the bulk of measures, but on the effectiveness of specific fiscal measures adopted by the authorities. Schindler et al. (2009) argue that the impact of fiscal measures on economic activity can be evaluated with the help of fiscal multipliers, defined as the ratio of the change in output in response to an exogenous change in a specific budgetary position. According to Batini et al. (2014), a fiscal multiplier measures the short-term impact of discretionary fiscal policy on output. There are various measurements of the fiscal multipliers, depending on the time horizon considered. In this context, different definitions can be found in the relevant economic literature: (i) impact multiplier or short term multiplier, (ii) multiplier at a specific time horizon, (iii) peak multiplier over any time horizon, and (iv) cumulative multiplier at a specific time horizon.

While the definition of fiscal multipliers and various time frame measurements seem to be widely accepted, there is little consensus in the literature on the size of multipliers. On the one hand, isolating the direct impact of fiscal measures on GDP is difficult given second-round effects and the twofold relationship between them. Researchers have tried to isolate exogenous fiscal shocks, namely those shocks that are not induced by the macroeconomic developments. Batini et al. (2014) note that there is no consensus regarding the methodology to identify such shocks. On the other hand, uncertainty over the size of fiscal multipliers also stems from limited data availability. The highest frequency for the budget execution according to the European System of Accounts (ESA) is quarterly data and, in case of some countries, this time series are not long enough to provide robust econometric estimations.

At the same time, empirical evidence confirms that there is no single fiscal multiplier. Hernández Hernández de Cos (2015) identify a series of factors highlighting the existence of state-dependent multipliers. For instance, given the composition of the fiscal shock, some government spending items are more likely to have higher short-run impact than government revenues. Furthermore, financial frictions, as well as nominal price and wage rigidities can lead to larger multipliers. When quantifying fiscal multipliers, the exchange rate regime (there are evidences for larger multipliers in the case of a fixed exchange rate regime) and the monetary policy reaction function (inactive monetary policy leads to higher fiscal multipliers) also play important roles. Another factor relates to the degree of openness of the economy, as a low degree of trade openness leads to a higher fiscal multiplier. Batini et al. (2014) identify similar factors influencing the size of fiscal multipliers. The determinants of the size are grouped in two categories: (i) structural country characteristics and (ii) conjunctural factors (namely, a series of temporary circumstances such as the cyclical position of the economy and the response of other policies to the fiscal shock). Within the conjunctural factors, the state of the business cycle is one of the main sources of differences among the size of fiscal multipliers. Baum and Koester (2011); Mittnik and Semmler (2012) or Auerbach and Gorodnichenko (2011) identify significant higher fiscal multipliers during recessions than in expansions. Glocker et al. (2019) also confirm that fiscal multipliers are state-dependent. Ilzetzki et al. (2011) argues that even though it seems to be widely accepted that fiscal multipliers are influenced by a series of factors and the business cycle is certainly among them, the size remains a source of controversy among economists.

In the case of Romania, there is also a lack of consensus regarding the size of fiscal multipliers. Empirical evidence differs depending on the size of the data sample and econometric tools used in estimations. Cozmanca and Voinescu (2020) find the cumulative expenditure multiplier to vary across 0.5 and 0.8 over the medium term and the revenue multiplier to be between 0.3 and 0.5. Lower revenue multiplier is found also by Voinescu (2018); Dumitrescu (2015) and Bobasu et al. (2013). Stoian (2012) estimates the revenue multiplier to pose slightly larger values than the expenditure one. At the same time, multipliers are found to be larger in booms compared to recessions and slowdowns. Nonetheless, according to Batini et al. (2014), in a comparison between fiscal multipliers in emerging market economies, their values are found to be positive and below 1. Overall, many of these studies list several caveats in their estimations.

In case of Poland, Czechia and Hungary, empirical evidence mostly finds relatively low values for fiscal multipliers, usually between 0 and 1. Szymanska (2019) estimated the 1-year cumulative spending multipliers for Czechia and Hungary at 0.4 and, respectively, 0.5. As regards Poland, empirical evidence showed a higher than 1 expenditure revenue, also confirmed by Derkacz (2020). By contrast, Batini et al. (2014) or Haug et al. (2019) estimate the spending multiplier in case of Poland to values close to 0.6.

## Stylized facts

In order to contain the spread of the SARS-CoV-2 virus, most countries introduced social distancing measures or even national lock-downs. Although the bleak picture of empty streets did not last too long, some sectors, especially those requiring high social interaction, suffered more than others and deep scars left by the pandemic can still be seen. Under these circumstances, the national authorities had a prompt response in limiting the adverse effects of the public health crisis.

Both the monetary and the fiscal policies have faced important reconfiguration. The packages of measures adopted in order to mitigate the negative effects of the crisis mainly relate to job retention, avoiding liquidity shortages and stimulating economic activity.

In the case of fiscal policies, countries adopted both expenditure and revenue support measures. According to IMF data, most of them relied on expenditure side, both in terms of healthcare and economic support. Significant funds were allocated to measures for job retention and assuring liquidity. These include: (i) payments for employment support (technical unemployment payments - the suspension of employment at the initiative of the employer as a result of a temporary deferral or reduction of activity, benefits for those returning to work), (ii) reduced-program schemes (for instance, *Kurzarbeit* schemes - social insurance programs that allow employers to reduce their employees’ working hours and wages instead of laying them off, while the government provides subsidies to at least partially compensate for diminished income), (iii) care allowance to parents, and (iv) care allowance to self-employed people.

Countries also adopted revenue side support measures. However, those were not as popular as the expenditure related ones. Most of them had zero budgetary impact, i.e. forgone revenue associated with the COVID-19 pandemic that partially started to be paid out in 2021 at the latest. Revenue side measures, having a budgetary impact referred to: (i) social insurance contributions covered by the public budget, (ii) discount for tax payments, and (iii) reduced VAT rate for various goods and services (especially in the case of medical products and those services substantially affected by social distancing measures).

**Fig. 1:**
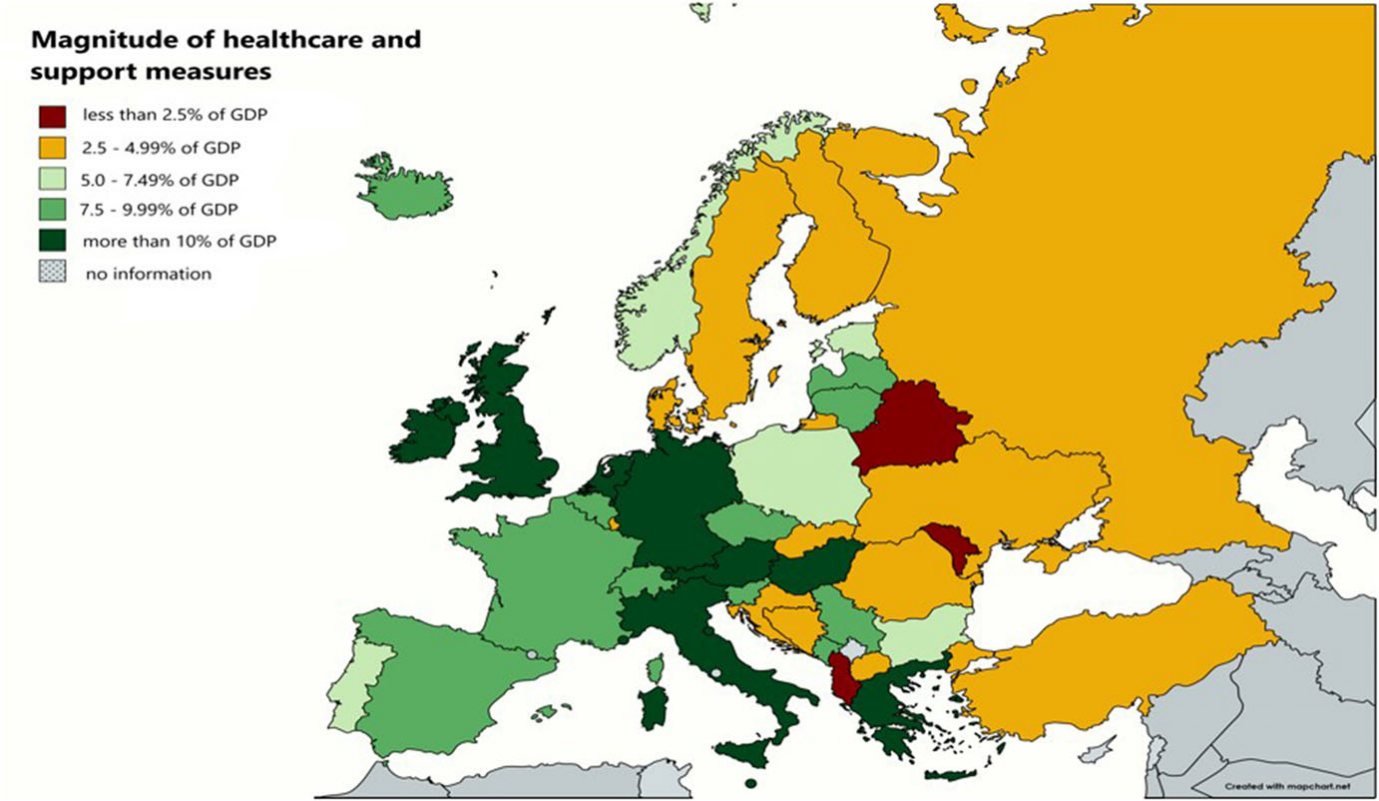
Additional Spending and Forgone Revenue in Response to the COVID-19 Pandemic as a share of GDP. *Source* IMF, authors’ calculations

**Fig. 2:**
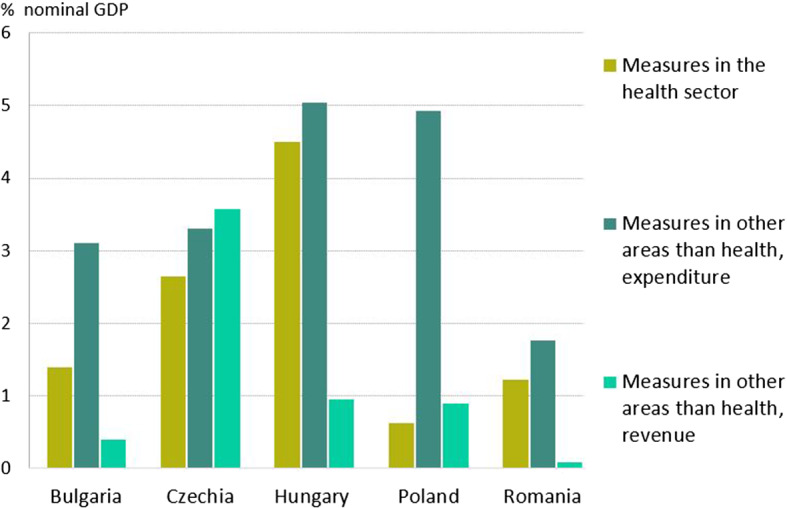
Support measures amount in selected CEE countries, % of GDP. *Source:* IMF, authors’ calculations

While most of the measures were synchronized among countries, the amount spent to limit the adverse effects of the coronavirus differs significantly, as shown in Fig. 1. In the case of CEE countries, namely Bulgaria, Czechia, Hungary, Poland and Romania, the most impressive package of support measures as a share of GDP was implemented in Hungary, while Romania was in the opposite side in these terms (Fig. 2). By the end of July 2021, Czechia and Hungary spent around 10% of GDP on support measures. In terms of additional expenditure in the health sector, the countries mentioned above also performed better. According to IMF estimates, the average COVID-19 health expenditure in case of the selected CEE countries is 2.1% of GDP, while the average economic support measures amounts to 4.8% of GDP.

**Fig. 3:**
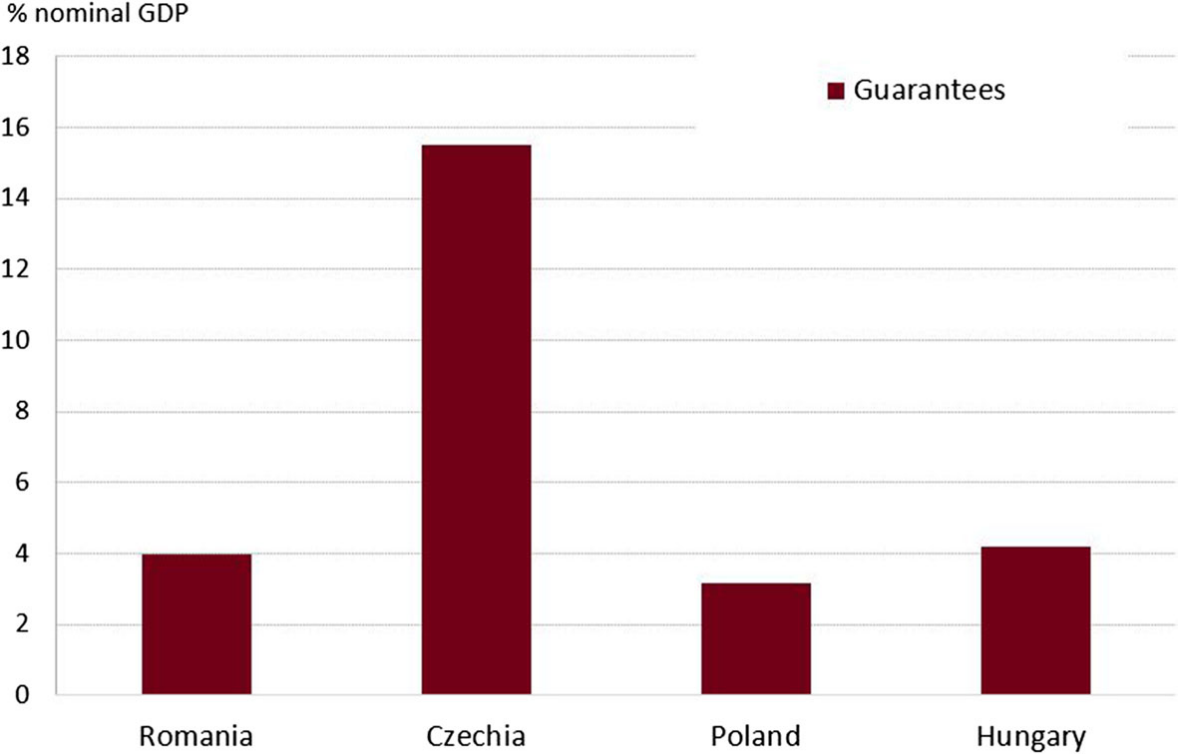
Guarantees in selected CEE countries, % of GDP. *Source:* IMF, authors’ calculations **Note:** No data is available in the case of Bulgaria in terms of guarantees

While most countries relied more on expenditure side measures, Czechia adopted an impressive revenue package. In addition to foregone revenue, the Czech authorities also introduced lump sums to be received by self-employed and the abolition of real estate transfer taxes. Furthermore, Czechia also outperformed in terms of guarantees, another form of government support (Fig. 3). This evolution relates to the introduction of several programs, such as: (i) the COVID Plus Program (state guarantees provided by the Export Guarantee and Insurance Corporation), (ii) the COVID II Program (state guarantees up to 80% of a loan, as well as a state contribution on interest costs), (iii) the COVID III Program (state guarantees up to 30% of loan principal), and (iv) other guarantees. Romania, Poland and Hungary also offered credit guarantees and micro-loans, especially to support the activity of SMEs, but of a lower value than in the case of Czechia. No data is available for Bulgaria regarding guarantees.

In addition to the response of national authorities, international institutions also played an important role in addressing the COVID-19 pandemic. For instance, the EU is mobilizing available resources to help member states coordinate their national responses. This includes, on the one hand, providing information about the spread of the virus and effective efforts to contain it and, on the other hand, measures to repair the economic and social damage brought by the pandemic. In this regard, international authorities decided that more flexible EU fiscal rules were needed and consequently the general escape clause was activated. Since its introduction in 2011, it has never been activated until the outbreak of the COVID-19 pandemic. This has allowed EU Member States to pursue a fiscal policy that facilitates the implementation of all necessary measures to address the public health crisis. In other words, they were allowed to record budget deficits above the threshold of 3% of GDP. However, in case of Romania, the Excessive Deficit Procedure (EDP) was activated in 2020 against the background of the escalated deficit from 2019. Despite the activation of the general escape clause, the EDP is still active in 2023 for Romania (yet a longer time for fiscal consolidation has been granted).

**Fig. 4:**
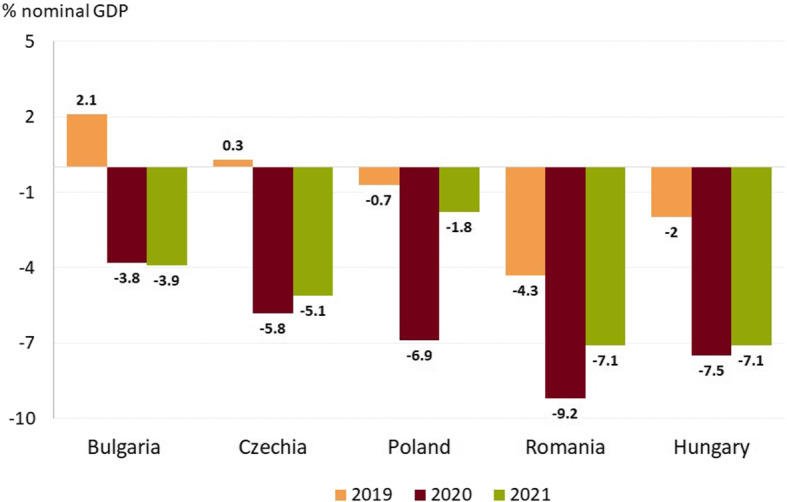
The budget balance (ESA 2010) in selected Eastern European countries. *Source:* EC, Eurostat, authors’ calculations *Note:* ESA refers to the European System of Accounts

According to Eurostat data, the budget deficit in European countries, including those in Central and Eastern Europe considered in this paper, has escalated rapidly amid both shrinking revenue and higher expenditure to help contain the virus and support economic activity (Fig. 4). In 2020, Romania recorded the highest ESA deficit, followed closely by Hungary. However, in the case of Romania, the expansionary fiscal policy stance before the outbreak of the coronavirus pandemic contributed to a deterioration of the public budget balance resulting in the activation of the EDP in 2020. In that year, spending related to COVID-19 and the contraction in public revenues led to a significant deficit. The deterioration of the balance compared to 2019 data is not the largest in this case, but for Poland, closely followed by Bulgaria.

The activation of the general escape clause was necessary given the outbreak of the novel coronavirus. The deterioration in the budget balance is expected to be only temporary, as support measures will fade and revenues could return to pre-pandemic levels. Given the protracted public health crisis, the EU’s fiscal rules also remain suspended in 2021 and 2022. Furthermore, the outbreak and extension of the war in Ukraine, which triggered an acute energy crisis and led to persistent bottlenecks in global value chains, determined the suspension of the general escape clause in 2023 as well. The risks to the budget balance outlook are rather downside, given the difficult socio-economic context at the current juncture. In this context, the implementation of new support measures to partly alleviate the effects of generalized high inflationary pressures is not excluded.

## Data and methodology

### Data

Government expenditure and revenues follow the definition used by Perotti (2004), which has been taken up in most studies in this field, as in Caldara and Kamps (2008) or Cozmanca and Voinescu (2020). The data source is Eurostat. More details, including data transformations, are available in Appendix 7.1. The time span analysed is between Q1 2000 and Q4 2021, however, shorter time intervals (starting with Q1 2003 or Q1 2008 and ending Q4 2021) are also considered to illustrate the intertemporal dynamics of the fiscal multipliers. In general, data before 2008 behave more volatile for some CEE countries, given the multitude of macroeconomic transformations that characterized the early 2000 s.

To make the transition from the shock-based representation (the main form of the models’ outputs) to the concept of fiscal multiplier, impulse response functions were processed to illustrate the impact of a monetary unit change (the response of GDP in EUR as a result of different shocks of one EUR at the level of fiscal variables), as in Blanchard and Perotti (2002) or Caldara and Kamps (2008). Considering different structural identifications, the results of the impulse response functions (which are expressed as percentages) are divided by the ratio of the fiscal variable to GDP. This ratio is evaluated as a unit value over the entire analysed period. In this procedure, the fiscal multiplier is defined as the response of GDP in currency units (EUR) to a fiscal shock of one monetary unit (EUR).

### Econometric models

This study uses several types of models to assess the level and the time dynamics of fiscal multipliers. Firstly, for a general analysis of the average multipliers during the entire time span a Bayesian Structural Autoregressive Vector (B-SVAR) model is employed. Secondly, in order to extend the degree of particularity to a higher level, a Time-Varying Parameter Structural Autoregressive Vector (TVP-SVAR) is used to capture the effects of fiscal policy measures on the real economy, period by period. In this way it is possible to assess the time variability of fiscal multipliers.

In the estimation of the B-SVAR model it is used an Independent Normal Wishart prior. In the case of this prior, unlike the Minnesota prior, the variance-covariance matrix of residuals is considered unknown, and unlike the Normal Wishart prior, it is assumed that the variance-covariance matrix of the coefficients can have an arbitrarily chosen structure, without the need for a linear relationship between residuals variance and coefficient variance. The easing of these hypotheses lead to a solution that could be found only by numerical simulation methods. The likelihood function in this case is illustrated in equation (1):

1 $$\begin{aligned} \begin{aligned} f\left( y \mid \beta , \Sigma \right) \propto \left| \det (\Sigma )\right| ^{-\frac{T}{2}}&\times \exp \left[ -\frac{1}{2}(\beta -{\hat{\beta }})^{\prime }\left( \Sigma \otimes \left( X^{\prime } X\right) ^{-1}\right) ^{-1}(\beta -{\hat{\beta }})\right] \\&\times \exp \left[ -\frac{1}{2} {\text {tr}}\left\{ \Sigma ^{-1}(Y-X {\hat{B}})^{\prime }(Y-X {\hat{B}})\right\} \right] \end{aligned} \end{aligned}$$

where *y* is a vector of *n* endogenous variables, $\beta$ a vector of parameters to be estimated, $\Sigma$ the variance-covariance matrix of residuals, *T* the size of the data sample, *X* a matrix of exogenous regressors, and ${\hat{B}}$ an estimate of the parameter vector *B*.

The prior distributions for parameters and residuals variance-covariance matrix are defined as in equations (2) to (5):

2 $$\begin{aligned} \beta\sim & {} N\left( \beta _{0}, \Omega _{0}\right) \end{aligned}$$

3 $$\begin{aligned} \pi (\beta )\propto & {} \exp \left[ -\frac{1}{2}\left( \beta -\beta _{0}\right) ^{\prime } \Omega _{0}^{-1}\left( \beta -\beta _{0}\right) \right] \end{aligned}$$

4 $$\begin{aligned} \sum\sim & {} I W\left( S_{0}, \alpha _{0}\right) \end{aligned}$$

5 $$\begin{aligned} \pi \left( \Sigma \right)\propto & {} \det (\Sigma )^{-\frac{(\alpha _{0}+n+1)}{2}} \times \exp \left\{ -\frac{1}{2} {\text {tr}}\left[ \Sigma ^{-1} \S _{0}\right] \right\} \end{aligned}$$

where $\pi (\beta )$ and $\pi (\Sigma )$ are the prior densities for $\beta$ and $\Sigma$, given $\beta _{0}$, $\Omega _{0}$ and $\S _{0}$, $\alpha _{0}$, respectively. The prior for $\beta$ is assumed to follow a multivariate normal distribution with mean $\beta _{0}$ and covariance matrix $\Omega _{0}$, which is an arbitrary matrix. The prior distribution for $\pi (\Sigma )$ is an inverse Wishart distribution, with scale matrix $\S _{0}$ and $\alpha _{0}$ degrees of freedom.

The results represent the conditional posterior distributions detailed in Eqs. (6) to (13).

6 $$\begin{aligned} \pi \left( \beta \mid \Sigma , y\right) \propto \exp \left[ -\frac{1}{2}(\beta -{\bar{\beta }})^{\prime } {\bar{\Omega }}^{-1}(\beta -{\bar{\beta }})\right] \end{aligned}$$

Equation (6) gives the kernel of a multivariate distribution with mean ${\bar{\beta }}$ and variance-covariance matrix ${\bar{\Omega }}$, computed as in Eqs. (7) and (8):

7 $$\begin{aligned} {\bar{\Omega }}= & {} \left[ \Omega _{0}^{-1}+\Sigma ^{-1} \otimes X^{\prime } X\right] ^{-1} \end{aligned}$$

8 $$\begin{aligned} {\bar{\beta }}= & {} {\bar{\Omega }}\left[ \Omega _{0}^{-1} \beta _{0}+\left( \Sigma ^{-1} \otimes X^{\prime }\right) y\right] \end{aligned}$$

So, Eq. (6) becomes:

9 $$\begin{aligned} \pi \left( \beta \mid \Sigma , y\right) \sim N({\bar{\beta }}, {\bar{\Omega }}) \end{aligned}$$

The conditional distribution for $\pi (\Sigma )$ can be written as in Eq. (10):

10 $$\begin{aligned} \begin{aligned} \pi \left( \Sigma \mid \beta , y\right) \propto&\left| \det (\Sigma )\right| ^{-\frac{\left[ \left( T+\alpha _{0}\right) +n+1\right] }{2}} \\&\times \exp \left\{ -\frac{1}{2} {\text {tr}}\left[ \Sigma ^{-1}\left[ (Y-X B)^{\prime }(Y-X B)+S_{0}\right] \right] \right\} \end{aligned} \end{aligned}$$

Now the kernel of the inverse Wishart distribution can be written as in equation (11), where ${\hat{S}}$ is the scale matrix and ${\hat{\alpha }}$ denotes the number of degrees of freedom, as detailed in equations (12) and (13), respectively.

11 $$\begin{aligned} \pi \left( \Sigma \mid \beta , y\right)\propto & {} I W({\hat{S}}, {\widehat{\alpha }}) \end{aligned}$$

12 $$\begin{aligned} {\hat{S}}= & {} (Y-X B)^{\prime }(Y-X B)+S_{0} \end{aligned}$$

13 $$\begin{aligned} {\hat{\alpha }}= & {} T+\alpha _{0} \end{aligned}$$

The Gibbs Sampling simulation algorithm is used to obtain the unconditional distributions of parameters and residuals covariance matrix starting from the two conditional posteriors.

In addition to the prior selection, another important element in both the B-SVAR and TVP-SVAR analysis is the type of structural identification. We used both triangular factorization and a sign restriction scheme. Triangular factorization is a general case of Cholesky decomposition, where the assumption that structural shocks have unit variance $(\Gamma \ne I)$ is relaxed. Therefore, in this situation $\Gamma$ is a diagonal matrix. The relationship between the reduced-form error variance-covariance matrix $\Sigma$ and the structural variance-covariance matrix $\Gamma$ can be written as in equation (14):

14 $$\begin{aligned} \Sigma =D \Gamma D^{\prime } \end{aligned}$$

In the case of this particular structural identification, as in the case of the Choleski decomposition, a special importance is given to the ordering of the variables in the model. Moreover, similar to the case of the Cholesky identification, in computing the posterior distribution of $\Sigma$, the matrix *D* is calculated together with $\Gamma$. These results are useful for constructing impulse response functions. For this type of structural identification, a shock to a fiscal variable represents a change of 1%.

The zero and sign restriction identification scheme is used as an alternative approach when quantifying income multipliers. Caldara and Kamps (2008) reason that in order to avoid expansionary fiscal contractions, namely a positive response of GDP to an increase in taxes, the best approach is this type of structural identification. Table 1 shows the zero and sign restrictions imposed on the estimations, along with the number of quarters they apply, in brackets.

**Table 1:** Identification scheme–the sign restrictions approach

	Business cycle	Prices	Net taxes	Monetary Policy
GDP	+(1–4)			
Inflation rate				
Net taxes	+(1–4)		+(1–4)	
Interest rate			0(1)	

*Source:* authors’ hypothesis

The TVP-SVAR model allows parameters to vary over time, having a specific set of results each period. They follow the autoregressive process described in equations (15) and (16):

15 $$\begin{aligned} \beta _{t}= & {} \beta _{t-1}+v_{t} \end{aligned}$$

16 $$\begin{aligned} v_{t}\sim & {} N(0, \Omega ) \end{aligned}$$

Thus, within the model there are 3 sets of parameters to be estimated: the coefficients of the VAR model (included in the vector $\beta _{t}$), the variance-covariance matrix of shocks from the autoregressive process ($\Omega$), and the variance-covariance matrix of residuals ($\Sigma$).

The B-SVAR and TVP-SVAR models are run in MATLAB using the BEAR toolbox developed by Dieppe et al. (2016).

## Results

### The effects of fiscal policy measures on GDP

#### B-SVAR results

The first model used is a B-SVAR, which shows the effects on GDP generated by a fiscal shock at the aggregate level of government expenditures or revenues, taking into account the entire time interval analysed. The data used for Figs. 5, 6, 7, 8, 9, 10, 11, 12, 13, 14 covers the range from 2008 Q1 to 2021 Q4.

**Fig. 5:**
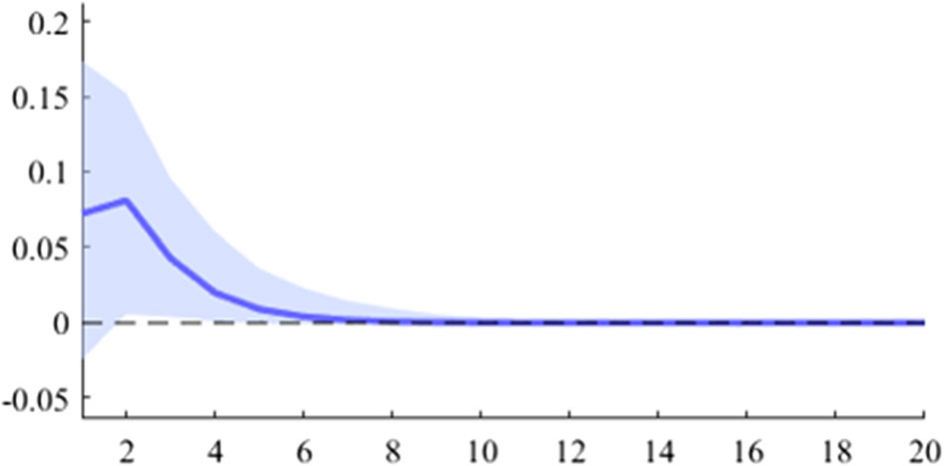
The GDP response to a 1% shock in government spending (Romania)

**Fig. 6:**
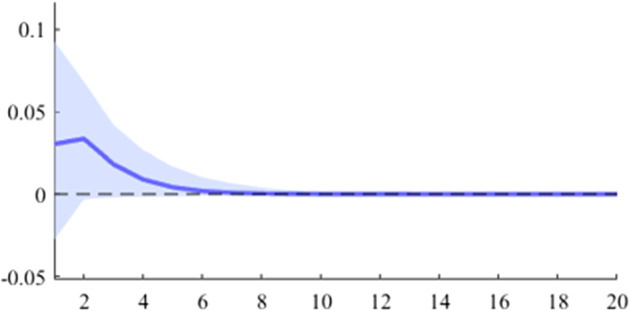
The GDP response to a 1% shock in government revenue (Romania)

**Fig. 7:**
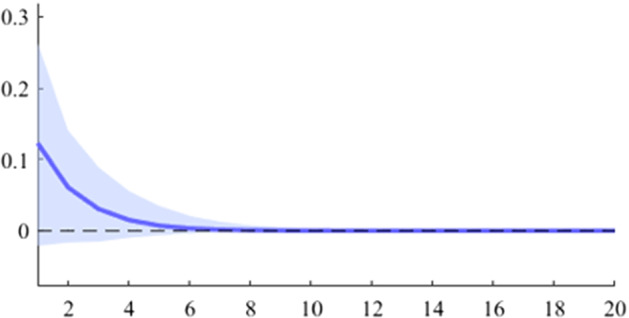
The GDP response to a 1% shock in government spending (Poland)

**Fig. 8:**
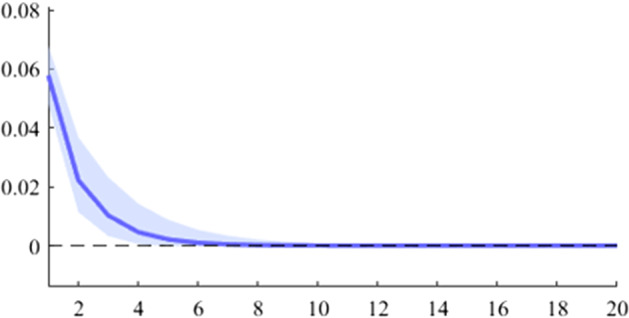
The GDP response to a 1% shock in government revenue (Poland)

**Fig. 9:**
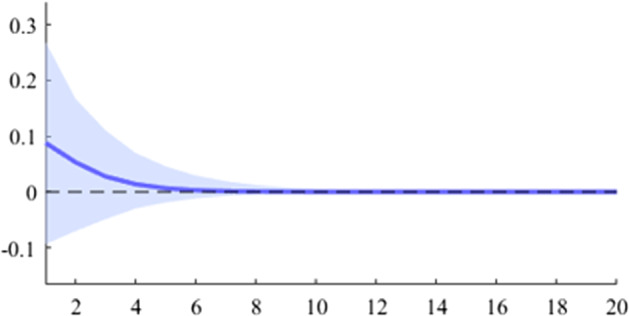
The GDP response to a 1% shock in government spending (Czechia). *Source:* authors’ calculations

**Fig. 10:**
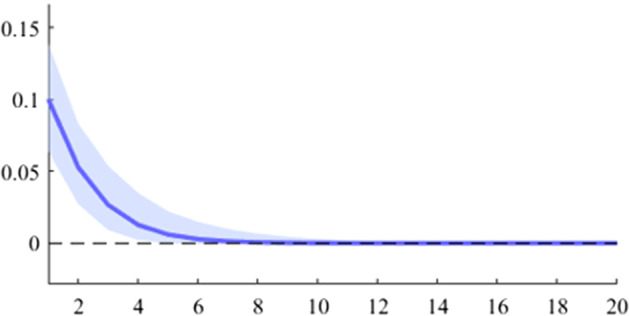
The GDP response to a 1% shock in government revenue (Czechia)

**Fig. 11:**
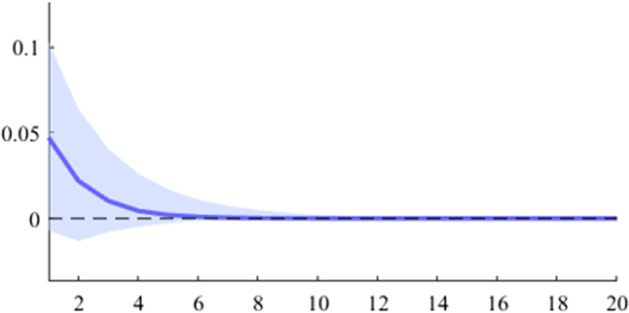
The GDP response to a 1% shock in government spending (Bulgaria)

**Fig. 12:**
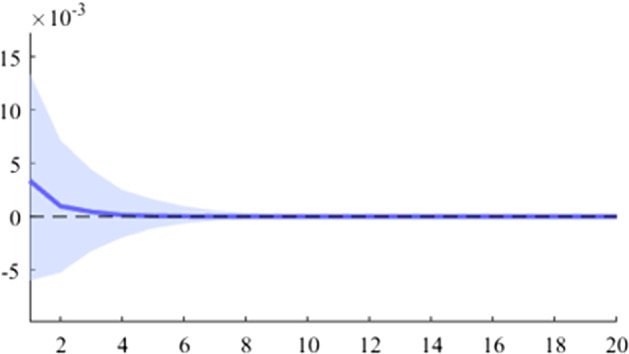
The GDP response to a 1% shock in government revenue (Bulgaria)

**Fig. 13:**
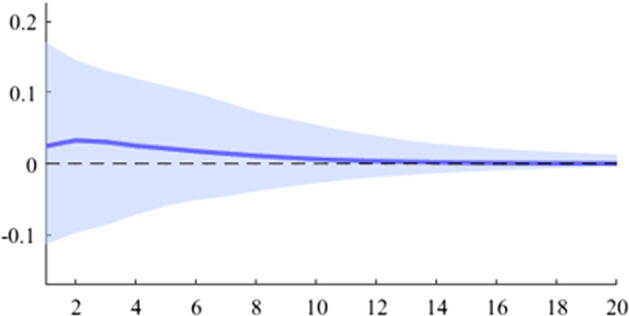
The GDP response to a 1% shock in government spending (Hungary). *Source:* authors’ calculations

**Fig. 14:**
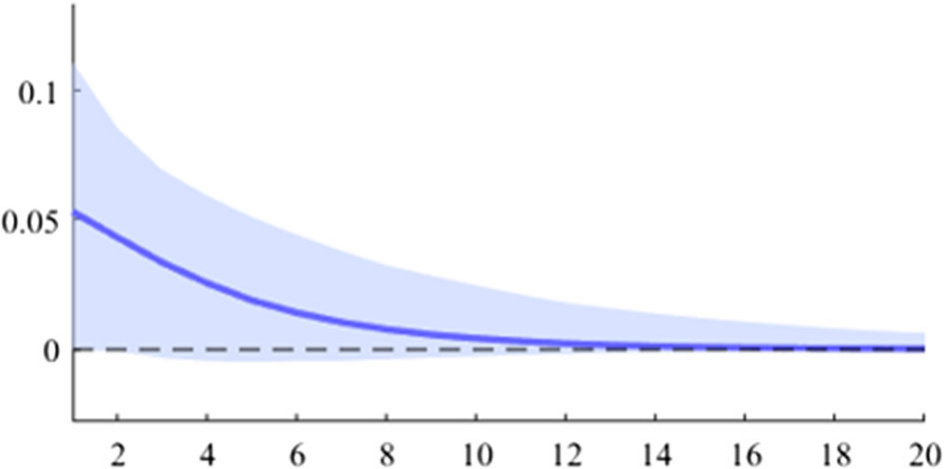
The GDP response to a 1% shock in government revenue (Hungary)

It can be seen that both in the case of shocks incurred at the level of government expenditure and at the level of government revenue, the GDP response is positive. When the identification scheme is based on sign restrictions, the economic activity response to government revenue becomes negative. However, depending on the economic structure of the countries, the response of GDP tends to be more or less statistically significant. The GDP response to government expenditure appears to be significant only in the case of Romania, while the response to the government revenue shock is significant in the case of Czechia and Poland.

Table 2 presents a detailed overview of government revenue and expenditure multipliers. In terms of government spending, the largest short-term fiscal multiplier was recorded in Poland, where GDP increases by about 0.6 units in a quarter after a one-unit shock. It is followed by Czechia and Romania, where the values are 0.45 and 0.37, respectively. Comparatively lower values were recorded in Bulgaria and Hungary, where the short-term spending multipliers were 0.26 and 0.11, respectively. Regarding the impact of the fiscal shock at one year horizon, only for two of the analysed countries the value of the fiscal multiplier is higher than one, namely in Poland and Romania. For the other 3 countries (Czechia, Hungary and Bulgaria), the impact of the fiscal shock is not fully transmitted in the economy even after 4 quarters.

**Table 2:** Fiscal multipliers through B-SVAR model

Multiplier	Country	Short term fiscal	Cumulative fiscal	Cumulative fiscal
		multiplier, (1 quarter)	multiplier (4 quarters)	multiplier (20 quarters)
	Romania	0.37	1.10	1.19
	Poland	0.61	1.12	1.19
Expenditures	Czechia	0.45	0.93	0.99
	Hungary	0.11	0.51	0.94
	Bulgaria	0.26	0.46	0.48
	Romania	0.20	0.60	0.65
	Poland	0.36	0.58	0.61
Revenues	Czechia	0.57	1.10	1.17
	Hungary	0.28	0.83	1.21
	Bulgaria	0.03	0.04	0.04

*Source:* authors’ calculations

The revenue multiplier appears to take positive values in all 5 analysed countries. The highest short-term values were recorded in the case of Czechia and Poland, followed by Hungary, Romania and Bulgaria. Regarding the long-term impact (20 quarters after the initial shock), it is observed that only Czechia and Hungary have a multiplier higher than one, while in Bulgaria the cumulative value of the long-term revenue multiplier is much lower.

Another important aspect to be mentioned is related to the value of the expenditure multiplier compared to the revenue multiplier. Thus, it is observed that in Romania, Poland and Bulgaria, the short-term spending multiplier is higher, while in Czechia and Hungary the situation is opposite. This result may provide insights into the direction in which the national policymakers should focus in the fiscal consolidation process.

As is commonly found in the literature, the revenue multiplier displays positive values, in contrast to economic theory. Auerbach and Gorodnichenko (2012) explain this phenomenon by highlighting two primary factors. Firstly, the identification of net taxes shocks requires separating discretionary measures from automatic responses to the business cycle (i.e. automatic stabilizers). Secondly, increased revenues may result not only from changes in taxation but also from improved collection. However, by estimating a BVAR model with zero and sign restrictions, we obtained negative revenue multipliers by imposing corresponding negative responses to revenue shocks.

Revenue multipliers are thus susceptible to measurement pitfalls. As previously elucidated, the most significant issue in this regard is the accurate identification of the pure fiscal shock. Firstly, changes in revenue variables are heavily influenced by output fluctuations and thus, these primarily constitute a policy effect rather than a policy tool. Secondly, policymakers often modify the revenue strategy in response to output fluctuations. These endogeneity issues are likely to distort the revenues multiplier figures. Moreover, another factor that can alter the size of the multiplier is that changes in revenues are likely to be anticipated by economic agents.

In an unrestricted SVAR model, the positive values for the revenue multiplier can be attributed to the issues highlighted in related literature, such as those discussed in Riera-Crichton et al. (2016). To filter out the automatic response of the government revenues to output fluctuations, a sign-restriction scheme can be imposed, such that a positive shock in the business cycle leads to an increase in government revenue. In contrast, no sign restrictions are imposed on the response of GDP (Table 1).

Table 3 exhibits the results obtained by the sign restriction approach. In Romania, the revenue multiplier seems to be somewhat higher, especially in the short term. At the same time, Bulgaria’s output is less sensitive to fiscal changes one quarter after the initial shock, but transmission increases significantly over the long term.

**Table 3:** Revenue multipliers - the sign restrictions approach

Multiplier	Country	Short term fiscal	Cumulative fiscal	Cumulative fiscal
		multiplier (1 quarter)	multiplier (4 quarters)	multiplier (20 quarters)
Revenues	Romania	$-$0.51	$-$0.63	$-$1.64
	Poland	$-$0.20	$-$0.37	$-$1.51
	Czechia	$-$0.40	$-$0.56	$-$1.59
	Hungary	$-$0.37	-0. 56	$-$1.31
	Bulgaria	$-$0.10	$-$0.18	$-$0.70

*Source:* authors’ calculations

#### TVP-SVAR results

The TVP-SVAR model is employed to estimate the impulse response functions in each quarter. This enables a more granular analysis of the impact of fiscal policy shocks on the real economy over the next 20 periods (5 years). Once again, in the lack of a sign-restriction identification scheme, the values for revenue multipliers turn positive, in line with the results previously obtained (more details are available in Appendix 7.2). As mentioned before, this evolution relates to a miss-identification of pure fiscal shocks.

**Fig. 15:**
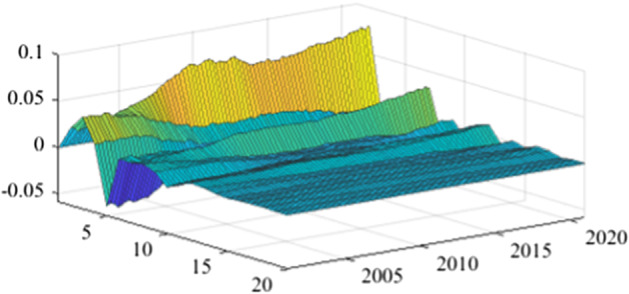
The GDP response to a 1% shock in government spending (Romania)

**Fig. 16:**
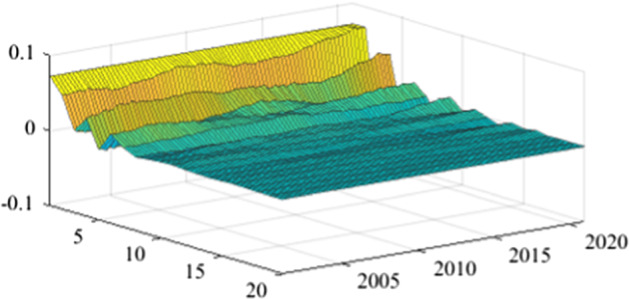
The GDP response to a 1% shock in government spending (Poland)

**Fig. 17:**
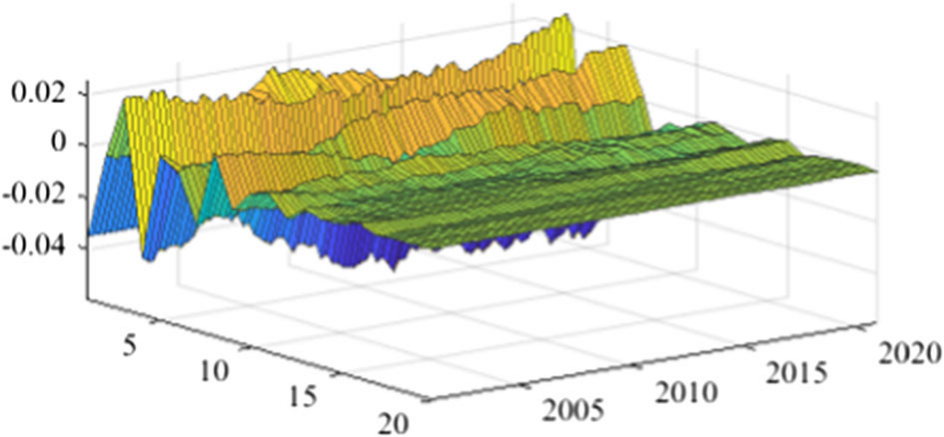
The GDP response to a 1% shock in government spending (Czechia)

**Fig. 18:**
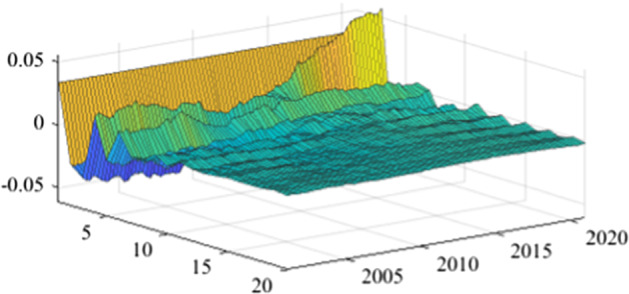
The GDP response to a 1% shock in government spending (Bulgaria). *Source:* authors’ calculations

**Fig. 19:**
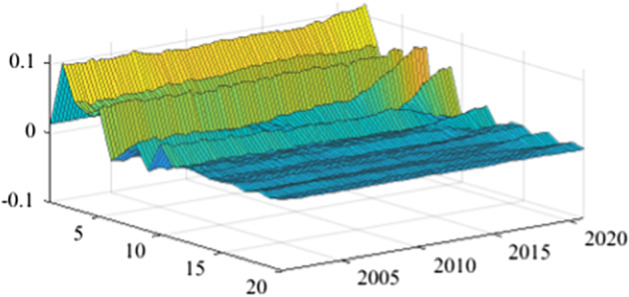
The GDP response to a 1% shock in government spending (Hungary)

Figures 15, 16, 17, 18, 19 demonstrate that the impact of an expenditure shock on real economic activity was felt differently at different time horizons within the analyzed period. Romania’s expenditure multiplier exhibits an overall upward trend, with a particularly strong increase in magnitude during difficult economic periods such as the last financial crisis and the COVID-19 pandemic. In contrast, Poland’s short-term expenditure multiplier remained relatively constant throughout the analysed period. Czechia and Bulgaria experienced similar trends to Romania, with multipliers increasing significantly during economic downturns compared to periods of growth. Finally, Hungary’s short-term expenditure multiplier increased sharply in the latter part of the sample, after 2018.

**Table 4:** The maximum values of the fiscal multipliers through the TVP-SVAR model

	Fiscal variable	Country	Short term fiscal	Iteration	
			multiplier (1 quarter)		
Maximum	Gov. spending	Romania	0.41	2021 Q1	
		Poland	0.61	2004 Q3	
		Hungary	0.52	2021 Q1	
		Czechia	0.13	2020 Q4	
		Bulgaria	0.30	2021 Q1	
Minimum	Gov. spending	Romania	0.10	2001 Q3	
		Poland	0.10	2017 Q2	
		Hungary	0.43	2009 Q2	
		Czechia	0.03	2012 Q3	
		Bulgaria	$-$0.25	2006 Q2	

*Source*: authors’ calculations

Table 4 shows the maximum and minimum values of the fiscal multipliers in the selected CEE countries for government expenditures. In Romania, the highest level of the spending multiplier was recorded in Q1 2021, while its minimum value was reached in the first part of the analysed interval (2001 Q3), yielding a variation between 0.10 and 0.41. In Poland, the highest value of the expenditure multiplier was recorded in the first part of the interval (2004 Q3) and the minimum value in the second part of it (2017 Q2). In Czechia, Bulgaria, and Hungary, the maximum values of short-term expenditure multipliers were recorded, similar to Romania, in the second part of the sample, more specifically during the COVID-19 pandemic, while the minimum values were recorded in 2009 Q2 and 2012 Q3. The corresponding fiscal revenues multipliers can be found in Table 9 of Appendix 7.2.

### The effects of fiscal measures on consumption and investment

In addition to the impact on gross domestic product, another important topic addressed in this paper is related to the effects of fiscal policy measures on private consumption and investment. The analysis was performed for the same five CEE economies (Table 5).

**Table 5:** Government spending fiscal multipliers for private consumption and private investment

Multiplier	Country	Variable	Short-term fiscal	Medium-term fiscal
			multiplier (1-4 quarters)	multiplier (8 quarters)
Government	Romania	Consumption	0.10	0.41
		Investment	0.30	1.11
	Poland	Consumption	0.35	1.86
		Investment	0.25	1.30
	Czechia	Consumption	0.04	0.15
spending		Investment	0.33	1.80
	Hungary	Consumption	0.03	0.13
		Investment	0.28	1.29
	Bulgaria	Consumption	0.03	0.15
		Investment	0.66	2.03

*Source*: authors’ calculations

In the case of Romania, the response of private consumption to a shock in government expenditure is lower than that of private investment in both the short and medium terms. Similarly, in Czechia, Hungary, and Bulgaria, private investment reacts more to such a shock than private consumption. Conversely, in the case of Poland, the fiscal multipliers for private consumption are higher than those for private investment; the short-term spending multiplier for consumption in Poland can reach 0.35, while in Hungary and Bulgaria it is significantly lower (0.03). With regard to private investment, the highest short-term spending multiplier was registered in Bulgaria (0.66), while the lowest was recorded in Poland (0.25).

### The effects of dis-aggregated government spending shocks

Given the recent pandemic and ongoing war in Ukraine, authorities have increasingly relied on specific fiscal expenditure measures in order to mitigate the negative impacts of these events. This sub-section focuses on the effects of these specific government expenditure categories. The three primary categories evaluated include: compensation of employees, investment expenditure, and social benefit expenditure. Drawing on the premise that aggregated government spending and revenue shocks have effects on the real economy, the analysis seeks to evaluate the degree to which these categories are effective in ameliorating the adverse economic impacts of the pandemic and war.

**Table 6:** The fiscal multiplier by categories of government spending

Multiplier	Country	Fiscal multiplier	Cumulative fiscal	Cumulative fiscal
		(1 quarter)	multiplier (4 quarters)	multiplier (20 quarters)
	Romania	0.2	0.4	1.5
	Poland	0.8	1.1	1.9
Compensation	Czechia	0.8	1.1	2.0
of employees	Hungary	0.7	0.9	1.8
	Bulgaria	0.3	0.5	2.1
	Romania	0.3	0.4	0.6
	Poland	0.7	1.0	2.0
Investment	Czechia	0.6	0.8	1.8
expenditure	Hungary	0.2	0.5	2.2
	Bulgaria	0.2	0.4	0.6
	Romania	1.0	1.2	2.1
	Poland	1.0	1.2	2.2
Social benefits	Czechia	0.8	0.9	1.5
expenditure	Hungary	0.8	1.0	1.2
	Bulgaria	0.3	0.4	1.9

*Source*: authors’ calculations

Table 6 documents the fiscal multipliers for different government spending categories for the five CEE countries analysed. Generally, the highest short-term fiscal multiplier tends to be the one associated with social benefits, followed by the one related to compensation of employees. However, Bulgaria displays comparatively low short-term fiscal multipliers for all types of expenditure. In contrast, Poland and Czechia exhibit multipliers above 0.6 for all categories, indicating a substantial economic response in the short term to an increase in any kind of government spending.

Over the longer term, spending on gross fixed capital formation yields higher multipliers in the case of more developed economies such as Poland, Czechia and Hungary, whilst in Romania and Bulgaria remain below 1 even at medium-term horizons. This evolution may be explained by a potential crowding out effect, as identified by Blanchard and Perotti (2002), who argue that increased government spending associated with large budget deficits can discourage private investment. Overall, the analysis indicates that in the case of all CEE countries the government expenditure can have a significant economic impact over the medium term.

### The effects of the fiscal measures taken during the COVID-19 pandemic

The assessment of the fiscal multipliers has a very important role in gauging the effect of fiscal policy measures on the real economy, especially during times of crisis such as the one caused by the emergence and spread of SARS-CoV-2 in 2020 and 2021. Such an instance raises the important question of the impact of the measures designed to support both firms and households and what would have been the economic decline in the absence of these measures. To address this, the initial step is to appraise the fiscal measures implemented to mitigate the effects of the COVID-19 pandemic (Table 7).

**Table 7:** The GDP impact of the fiscal measures adopted during the COVID-19 pandemic

Country	Exceptional expenditure in the	Spending	Impact on nominal GDP generated	GDP for 2020
	context of COVID-19 pandemic*	Spending	by fiscal measures	
Romania	EUR 6.78bln	0.40	EUR 2.71bln	EUR 218.86bln
Poland	EUR 33.51bln	0.16	EUR 5.27bln	EUR 523.66bln
Hungary	EUR 14.34bln	0.50	EUR 7.22bln	EUR 136.62bln
Czechia	EUR 20.66bln	0.12	EUR 2.48bln	EUR 215.25bln
Bulgaria	EUR 3.06bln	0.29	EUR 0.89bln	EUR 61.33bln

$^*$ The amount does not contain guarantees but it consists of the discretionary measures with a budgetary impact granted by national authorities in order to alleviate the effects of the COVID-19 pandemic

*Source:* authors’ calculations based on data from the National Ministries of Finance, Eurostat and IMF

Among the five analysed countries, Hungary implemented the most consistent fiscal package, amounting to 10.5% of nominal GDP. This was mirrored by a relatively higher value of the spending multiplier, thus indicating a relatively better efficiency in the allocation and use of government spending. Czechia allocated a significant 9.6% of nominal GDP in support measures; however, the value of the spending multiplier was relatively smaller, suggesting a potential lower impact on the real economy. Poland’s fiscal package was estimated to 6.4% of GDP, benefiting from a significant fiscal multiplier in both the short and medium term. Romania and Bulgaria deployed relatively lower fiscal packages, of 3.1% of GDP and 5% of GDP, respectively; however, given the increase in fiscal multipliers in recent years in these countries, the impact of the measures on the real economy is likely to be significant.

## Concluding remarks

The COVID-19 pandemic proved to be an unprecedented socio-economic crisis, prompting swift and decisive action from both national and international authorities to mitigate its economic impacts. In response, the European Commission activated the general escape clause within the Stability and Growth Pact for the first time, allowing European countries to run high deficits starting with 2020. The measure was also extended for the year 2023 in the context of the energy crisis accentuated by Russia’s invasion of Ukraine. Under these circumstances, impressive fiscal support packages were adopted. Our research reveals that, while the magnitude of these measures varied significantly across CEE countries, the instruments for alleviating adverse effects were largely similar, and have proven effective in containing the economic contraction.

In selected Central and Eastern European (CEE) countries - Bulgaria, Czechia, Hungary, Poland, and Romania - the magnitude of fiscal stimulus packages adopted in response to the COVID-19 pandemic between its outbreak and 2021 ranged from approximately 3.1% of GDP in Romania to 10.5% of GDP in Hungary. Apart from Czechia, the selected countries largely implemented expenditure-side measures, such as subsidies for furloughed workers and reduced-time employment schemes.

In this analysis, the size of fiscal multipliers is evaluated in order to assess the efficiency of individual measures. Empirical evidence suggests a greater GDP response to expenditure shocks, with estimated fiscal multipliers within a quarter ranging from 0.03 in Czechia to 0.61 in Poland, values that approach or exceed one on medium-term in all countries except Bulgaria. Conversely, results indicate that multipliers for revenues are lower than those for government expenditure.

Empirical evidence suggests that there are difficulties associated with the measurement of revenue multipliers. A sign-restriction identification scheme was implemented in order to identify the automatic response of revenue variables to output fluctuations and yielded negative multipliers, as expected. These results suggest that further research should be conducted to explore alternative methods for the identification of a pure fiscal shock. For instance, a narrative approach could be employed to construct a new revenue series based on specific policy instruments.

In the related economic literature, the size of fiscal multipliers is a matter of debate. However, the sensitivity of fiscal multipliers to economic cycles is an accepted characteristic. To assess the impact of the COVID-19 pandemic on fiscal multipliers, a time-varying B-SVAR model was employed. The empirical results indicate an intensification of both expenditure and revenue multipliers during the economic crisis.

In light of our results and knowing the composition of the fiscal support packages, the impact of such measures on economic growth was estimated. The prevailing use of expenditure side measures in tandem with further amplifications in the size of fiscal measures is assessed to contribute to a lower contraction in 2020 and a speedier recovery in 2021 by almost 2 percentage points, on average at the level of the 5 analysed CEE countries. Empirical evidence suggests that this emphasis on expenditure-side measures over revenue-side ones was a beneficial choice, as expenditure multipliers tend to be higher. In the upcoming period, economic recovery should further rely on similar measures.

To sum up, the measures implemented by authorities proved to be highly effective. Nevertheless, had they been aware of fiscal multipliers of certain expenditure and revenue items, their positive impact could have been maximized. Taking into account the unstable socio-economic situation in CEE region given the ongoing war in Ukraine, new support fiscal measures could be implemented. The empirical evidence in this paper can be used to evaluate their efficiency. Although this results provide useful information for policymakers, we are aware of their limitations. As previously noted in the literature review section, the size of fiscal multipliers is influenced by a series of structural and conjunctural factors. A more granular approach could be a subject of further research. For instance, investment expenditure could be disaggregated by the source of financing (domestic or foreign) or by specific sectors. Moreover, with the implementation of the recovery instrument Next Generation EU already in progress, more evidence regarding the impact of EU funds could be an interesting research topic.

## Figures and Tables

**Fig. 20:**
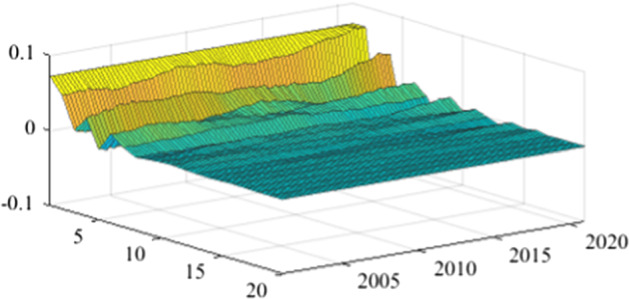
Romania

**Fig. 21:**
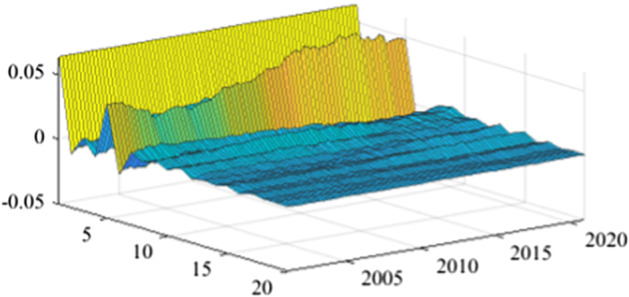
Poland

**Fig. 22:**
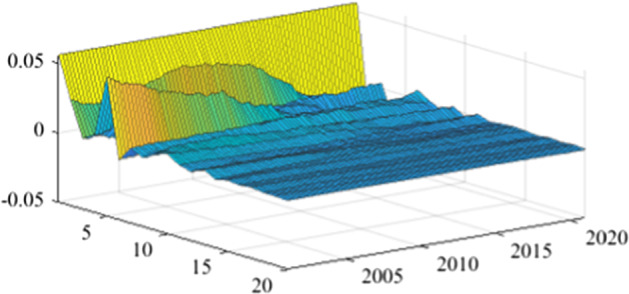
Czechia

**Fig. 23:**
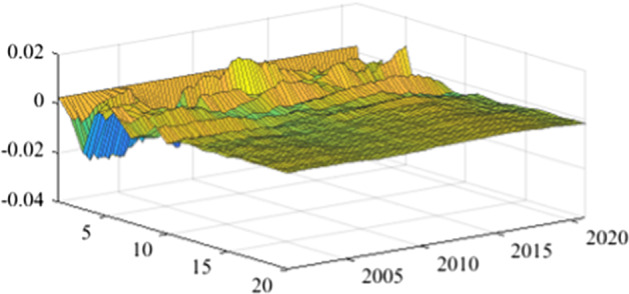
Bulgaria. *Source:* authors’ calculations

**Fig. 24:**
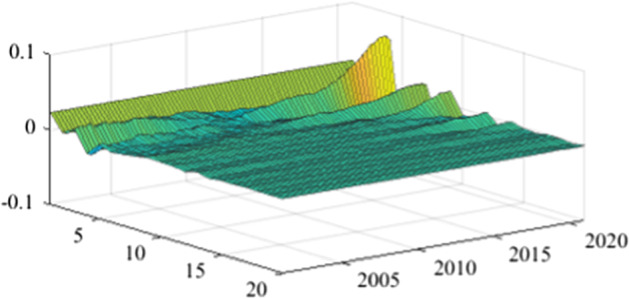
Hungary

## 7 Appendix

### Data

Table 8.

**Table 8:** Data processing

	Description	Definition	Data transformation	Source
Y	Gross domestic product	Chain linked volumes, percentage change compared to the same period in previous year (million EUR)	seasonal adjusted, real values, per capita, logarithm operator, differentiation, annualized	Eurostat
G	Government expenditure = Compensation of employees + Intermediate consumption + Gross fixed capital formation	Current prices (million EUR)	seasonal adjusted, real values, per capita, logarithm operator, differentiation, annualized	Eurostat
R	Government revenues = Revenues from direct taxes + Revenues from indirect taxes + Revenue from social contributions - subsidies transfers	Current prices (million EUR)	seasonal adjusted, real values, per capita, logarithm operator, differentiation, annualized	Eurostat
Cp	Private consumption	Current prices (million EUR)	seasonal adjusted, real values, per capita, logarithm operator, differentiation, annualized	Eurostat
Ip	Private investments	Current prices (million EUR)	seasonal adjusted, real values, per capita, logarithm operator, differentiation, annualized	Eurostat
Cs	Compensation of employees	Current prices (million EUR)	seasonal adjusted, real values, per capita, logarithm operator, differentiation, annualized	Eurostat
CI	Investment expenditure	Current prices (million EUR)	seasonal adjusted, real values, per capita, logarithm operator, differentiation, annualized	Eurostat
CBS	Social benefits	Current prices (million EUR)	seasonal adjusted, real values, per capita, logarithm operator, differentiation, annualized	Eurostat

### 7.2 TVP-VAR results: the GDP response to a 1% shock in government revenue

See Figs. 20, 21, 22, 23, 24 and, Table 9.

**Table 9:** The maximum values of the fiscal multipliers through the TVP-SVAR model

	Fiscal variable	Country	Short term fiscal multiplier (1Q)	Iteration	
Maximum	Revenues	Romania	0.47	2020 Q2	
		Poland	0.11	2010 Q1	
		Hungary	0.02	2006 Q4	
		Czechia	0.22	2008 Q3	
		Bulgaria	0.07	2011 Q3	
Minimum	Revenues	Romania	0.29	2004 Q2	
		Poland	$-$0.13	2020 Q4	
		Hungary	$-$0.09	2016 Q2	
		Czechia	$-$0.18	2021 Q1	
		Bulgaria	$-$0.14	2002 Q4	

*Source*: authors’ calculations
